# The Responsibility of the Examining Physician of the Insane, Particularly with Reference to Their Commitment to Asylums*Read before Alumni Association of Buffalo Medical College, Feb. 27, 1883.

**Published:** 1883-04

**Authors:** P. M. Wise

**Affiliations:** Assistant Physician Willard Asylum for the Insane


### The Responsibility of the Examining Physician of the Insane, Particularly with Reference to their Commitment to Asylums.*

By P. M. Wise, M. D.,

Assistant Physician Willard Asylum for the Insane.

Mr. President—I trust it will not be deemed inappropriate if I call your attention, briefly, to a subject that is not purely scientific, but, it appears to me, of sufficient importance for your consideration.

The examination of reputed insane persons with a view to their commitment to lunatic hospitals or asylums, either for treatment or custodial care, or both, is an important function of the practicing physician. He holds, in this respect, a unique and peculiar relation to the community. He exercises not only a purely medical function in the diagnosis of an alien disease, but, in the State of New York, as well as in several neighboring States and in Canada, certifies as well to the propriety of asylum commitment, upon which depends, or to which certificate is appended a judicial approval or order.

My subsequent references will apply to the commitment of the insane in the State of New York by civil process under the

* Read before Alumni Association of Buffalo Medical College, Feb. 27, 1883.

existing statutes. Briefly, then, under this process no person can be committed to a receptacle for the insane except upon the certificate of two physicians, who certify, under oath, to their qualifications as medical examiners, and after a personal examination setting forth the insanity of such person. Upon these certificates alone a subject can be provisionally committed to an asylum pending judicial action, for a period not exceeding five days. It is well to understand that this grant of power constitutes the chief legal responsibility of the examiner. To quote from an opinion of the ex-Commissioner of Lunacy: * "The physician who is permitted to exercise it should remember that, like all legal franchises, he takes it *cum onere.* Hence an action will lie against him for maliciously, and without any reasonable or just cause, signing a certificate that a party was insane and in a state requiring to be confined, in consequence whereof a party had been detained in custody as a lunatic. Or, such certificate might be considered a libel, in which case an indictment would lie against the person signing it. Therefore, our courts have held that it is clearly libelous to publish of another that he is 'insane and a fit person to be sent to the lunatic asylum,' or that 'he is so disordered in his senses as to endanger the persons of other people if left unrestrained, and that it is dangerous to permit him longer to go at large.' The libelous character of such language will not be destroyed or diminished by the fact that the person uttering it is a physician and makes the statement as a professional opinion."

A medical certificate becomes of full force only after receiving the approval of a judge or justice of a court of record; but the responsibility still rests upon the physician who has furnished not only the facts, as observed by himself, but has certified to the propriety of detention in an asylum under the provisions of the statute.

The form of the certificate is prescribed by the Commissioner of Lunacy, and the one in force at the present time is specified in

* Ordronaux: "Judicial Aspects of Insanity."

the appendix of the codified lunacy laws of 1874. It is the same schedule that is prescribed by the English lunacy laws, except in one particular to which I shall hereafter refer.

You are undoubtedly familiar with the form of certificate, but I ask your attention to a suggestive review of its requirements: The schedule provides for the name, the residence and the legal qualifications of the examiner; for the statement, under oath, that on a stated day a personal examination of the party named had been made, and following that the opinion that the said party was insane and a proper person for care and treatment under the provisions of the statute. The declaration of insanity is an entirely distinct one from the succeeding statement that the person is a proper one to be detained for care and treatment. This is a question that may at some time require your serious attention; but as the certificate in question is always made with reference to the admission of the patient into an asylum, the examiner must be competent to attest the propriety of such action.

Although it may be negatively held that a person of unsound mind is not, legally, an improper one for admission to an institution specially designed for their care, it must be remembered that in very many cases attesting such an opinion places the examiner in a defensive position, and, in a greater degree than in ordinary professional opinions, he may find it necessary to justify his action afterwards.

In this connection I deem it proper to refer to some of those conditions of insanity in which, as medical advisers of the family of the lunatic, the physician may be called upon to decide as to their future treatment. The propriety of asylum commitment may be submitted to him, or it may be opposed by the family in instances where it appears to him an urgent necessity. The judgment of the physician, however, usually prevails. In this capacity he assumes a responsibility not legal in its character but essentially grave. To illustrate, he may be called to a case of acute mania with delirium that has suddenly developed and has thrown the immediate friends into a state of utmost confusion and despair. They look to the doctor for relief and advice, and he, perhaps with the consciousness of an incapacity to comprehend the requirements of the situation, advises immediate commitment to an asylum. Within a week the patient may die, as he would have done at home or under domestic care, but the doctor's professional acumen has to bear, none the less, the criticism, and sometimes the contumely, of the friends.

On the other hand, his judgment is enlisted in a case of mild progressing mania. The patient may not be a disturbing element in the household, and examination reveals, probably, a delusion of persecution, with perhaps illusions of sight or hearing. There may be strong opposition in the family to removal from home, and the doctor, aware that his decision will govern action in the case, concludes to advise continued residence in the family. Or, what appears to him a mild form of melancholia with depressing apprehensions may not be considered urgent, and he carelessly recommends suspension of action, and the patient continues at home. A tragedy following in either case, would tend towards making the medical adviser feel very uncomfortable, and he would undoubtedly appreciate the responsibility that attends his professional calling at that time as much as at any other in the course of his life.

It may be laid down as a pretty safe rule to follow—not one of course, without frequent exceptions—that cases of acute delirious mania should be retained at home until it is safely conclusive that the attack is not a transient one, and that the patient will survive it. Marcé, a well-known French writer estimates that one out of three or four survive the attack. In this country the mortality is certainly not as great, but it is large and it is well to bear this in mind in making a prognosis that will largely influence the disposal of the patient. And then again, it is well to act slowly in these cases until the diagnosis is settled. The patient may be drunk, in a condition of delirium tremens, or suffering from delirium of acute disease. Moreover, they can be as successfully treated at home if proper care can be given them, the chief requirement being constant and sufficient personal attention.

I cannot, in such a brief paper refer to the various conditions of insanity requiring asylum care, but I would especially impress upon you that patients with homicidal and suicidal tendencies, as a matter of safety, need the watchful care that is best given them in asylums.

Entirely aside from the question of treatment, it is best to provide the protection of an asylum for a patient with active delusions, particularly if they are delusions of persecution or are of a depressing character and lead to apprehensions or the baser passions. In cases attended by mental depression there should always be a fear of suicide, and where proper vigilance cannot be afforded at home it is the doctor's function, in so far as his counsel will prevail, to see that it is afforded in an asylum. Reproach will be deserved if the physician allows the popular clamor against asylums to influence his counsel in cases that are manifestly dangerous to themselves or others.

Then, again, there are conditions of chronic mania known in the nomenclatural multiplication table as pyro-mania, eroto-mania, demono-mania, nympho-mania, etc., that are not harmless conditions, and that require the restriction of liberty given with the least friction in a well-conducted asylum.

Upon the family physician frequently devolves the responsibility of seeing and treating the insane individual in the only curable stage of the disease, and it is an obligation he owes his calling that he either adopt and apply such remedial measures as the case requires, or else inform the friends in no uncertain way that the danger of confirming the insanity lies in the postponement of treatment.

With this digression, I again ask your attention to the legal form of the certificate of insanity. The schedule requires the examiner's opinion, as already stated, but it also requires the reasons or grounds, which should be facts, upon which such opinion is formed. The defective quality of certificates is usually found in the imperfect narration of facts. It should be plain that the reasons upon which an opinion is based should not be inconsistent with sanity, but should plainly set forth the observed facts that lead the examiner to his opinion, as well as the leading incidents communicated by others that tend to confirm such an opinion.

In the schedule of the British certificate, in that of Ontario and several of the States, the reasons are divided in the form in two parts: 1st. As to facts indicating insanity observed by the examiner. 2d. Other facts (if any), indicating insanity, communicated by others. It is better to state the result of the personal examination of the patient independent of the information derived from other sources, whether such a division is required by the form or not; for testimony of this character is of greater forensic value than vague generalizations. It is usually the only testimony that the judge who approves the certificate has, and it would be reasonable and just were he to demand, in each instance, satisfactory proof of the insanity of the party from the facts observed by the examiner.

I quote the following reasons given in a few of the certificates that have come under my observation, to illustrate the value of such a distinction:

"From a personal examination and the history, I am convinced that he is afflicted with insanity." The examiner merely re-states what he has previously stated in the certificate.

"That his imperfect nervous development assumes a form of maniacal insanity."

"Having been her medical adviser for two years, am well conversant with her condition." It would seem that a two years' acquaintance with the party ought to make the examiner competent to state a few relevant facts.

"Upon a personal examination, and having been acquainted with him for nineteen years."

"I have seen her almost daily for several years, and from the fact that she has been once before in an asylum." It may be a satisfaction to the court to know of these long acquaintances, but these are surely not facts indicating insanity.

"She has a difficulty of the uterus, a difficulty of the spine in the dorsal region, perhaps organic." The author of this certificate may have presumed there was sufficient peripheral irritation to constitute a case of reflex insanity, but he withholds his theory.

Another examiner certifies in several cases, with marked uniformity, that the fact that grounded his opinion was "Chronic Dementia," whereas, it was a fact, in one of the cases at least, that dementia was the least noticeable symptom.

"That she is in a state characterized by an association of non-related perceptions from inability of the mind to judge and reason."

"Rapidity of the pulse, restlessness, general debility and inconsistent conversation. Thinks he is dangerously sick." The companion certificate, made by a disciple of Hahnemann, was also based upon the ground that the party considered his own case dangerous. As a matter of fact, the patient manifested many symptoms of insanity and expressed delusions of grandeur freely.

Now, as regards facts, whether observed or communicated, there were none at all in the certificates quoted that were inconsistent with the patient being perfectly sane.

When a physician undertakes to examine a person for the purpose of signing a certificate, he has to decide whether the person is or is not of unsound mind. If the latter opinion is reached, he should be able to plainly state the reasons for it.

The examination of insane persons is frequently attended by difficulties, various in kind and degree. In urgent cases of insanity, especially in the acute form, the manifestations of disease are easily recognized, but there are many insane conditions where the indications of a mental disorder are not plainly apparent to the examiner upon a personal examination, and he is, therefore, incompetent to sign a certificate, however well he may be convinced by the information communicated that the party is insane. In cases of doubtful insanity and so-called "moral insanity," in minds that have left the realm of eccentricity and have drifted over the borderland, but where sufficient mental integrity remains to permit a temporary restraint of the diseased exhibit, the examiner may encounter the greatest difficulties and be seriously baffled. Frequently there are no delusions or hallucinations, but only intellectual defect. The mind, by original organization of the brain, may be deficient and incompetent, and this insufficiency of mental control may increase to such a degree as to render the subject irresponsible for his acts and a proper person for supervision and restraint; but the examiner does not see the insane conduct, as it is only exhibited under commonplace conditions, and they are frequently able to defend their insane acts by creditable arguments. The examiner should be satisfied that the insane action he may observe, is of a persistent and not of a fugitive character, or a transient condition. There are many phases of erratic action, of violent passion and eccentric states of the intellect that would constitute serious forms of insanity, were they persistent and not explained by the environment of the individual.

Before entering upon the personal examination of difficult cases, the examiner should prepare himself with all the presumptive evidences of insanity at his command, and thus be provided with a base for operation, and a starting point. An ingenious introduction of an examination frequently affords the physician opportunities that, by an awkward introduction he would not obtain in many interviews. As a rule, the insane are easily led to talk about their habits of life, and if they have delusions they are usually connected with the practices of every-day life. The examiner must accept the statements of friends in an impartial manner and consider such evidence as merely corroboratory, to be proved by observed facts. It must also be remembered that one symptom of insanity, one insane act, is not enough to prove its existence.

The difficulty of the examiner may arise when he endeavors to define the abnormal characteristics of these perplexing cases upon his certificate as grounds for his opinion; to draw from obscurity to plain description his consciousness of the morbid condition. It may be his sad experience to support the views expressed in his certificate on the witness stand, for it is these very cases that furnish the material for courts.

And now, gentlemen, in closing this brief and imperfect treatment of so important a subject I desire to express the hope, which I am sure is equally shared by you, that our alma mater may continue to advance her standard of excellence; and that she may add to the ably-filled chair on insanity some opportunities for the clinical teaching of this important department of medicine. It is but a few years since the study of mental disease has been added to the curriculum of any American school, and we may well feel proud of our foster-mother that she holds her position in the advance column. May we not hope that she will perfect the good work so auspiciously begun in this direction by adding a clinic in psychiatry.

